# The Biocontrol Potential of Endophytic *Trichoderma* Fungi Isolated from Hungarian Grapevines, Part II, Grapevine Stimulation

**DOI:** 10.3390/pathogens12010002

**Published:** 2022-12-20

**Authors:** András Csótó, Csilla Kovács, Károly Pál, Antal Nagy, Ferenc Peles, Erzsébet Fekete, Levente Karaffa, Christian P. Kubicek, Erzsébet Sándor

**Affiliations:** 1 Institute of Plant Protection, Faculty of Agricultural and Food Science and Environmental Management, University of Debrecen, H-4032 Debrecen, Hungary; 2 Kálmán Kerpely Doctoral School, University of Debrecen, H-4032 Debrecen, Hungary; 3 Research Institute Újfehértó, Agricultural Research and Educational Farm, University of Debrecen, H-4244 Újfehértó, Hungary; 4 Institute of Food Science, Faculty of Agricultural and Food Science and Environmental Management, University of Debrecen, H-4032 Debrecen, Hungary; 5 Department of Biochemical Engineering, Faculty of Science and Technology, University of Debrecen, H-4032 Debrecen, Hungary; 6 Institute of Metagenomics, University of Debrecen, H-4032 Debrecen, Hungary; 7 Institute of Chemical, Environmental & Bioscience Engineering, TU Wien, A-1060 Vienna, Austria

**Keywords:** *Trichoderma*, grapevine, rootstock soaking treatment, fungal colonization of plants

## Abstract

In the first part of this two-piece publication, the isolation, identification and in vitro characterization of ten endophytic *Trichoderma* isolates were reported. Here we report the ability of two different mixes of some of these isolates (*Trichoderma simmonsii, Trichoderma orientale* and *Trichoderma gamsii* as well as of *Trichoderma afroharzianum* and *T. simmonsii*) to colonize and stimulate the growth of grapevines. Two commercial vineyards about 400 km away from the site of isolation were used as experimental fields, from which the strains of three *Trichoderma* species were re-isolated up to four years after rootstock soaking treatment with conidiospores, performed before planting. The treatments decreased the overall percentage of lost plants of about 30%, although a low number of lost plants (about 5%) were observed also in the control plot. For all cultivars and clones, the *Trichoderma* treatments significantly increased both the bud burst ratio and bud burst vigor index. In addition, the grape must parameters such as the Brix degrees, as well as the extract, the D-glucose and the D-fructose concentrations all appeared to be improved, suggesting a potentially higher ethanol content of the produced wine. We conclude that grapevine-endophytic *Trichoderma* isolates promote plant growth, which could be a useful feature for sustainable agriculture in general and integrated plant production in particular.

## 1. Introduction

Microbes whose presence is beneficial for plants may act as (i) biostimulants, (ii) inducers of plant defense/resistance mechanisms, and (iii) antimicrobial effect against pathogens [1]. This triple mode of action has been demonstrated for *Aureobasidium pullulans*, *Clonostachys rosea* and *Trichoderma* spp. among Ascomycetous fungi [2,3,4].

*Trichoderma* species are widely known for their mycoparasitic and antagonistic properties [5,6,7]. Some strains are also able to stimulate plant growth and development [5,8,9], and to increase stress tolerance [10,11]. Enhanced root growth was observed in maize, cucumber, pea and tomato [12,13,14], while both root and shoot growth were significantly increased in sweet cherry by *Trichoderma* [9]. The expansion of shoot length and shoot diameter in vegetables was also reported [11,15]. The higher number of leaves and larger leaf surface area, as well as the higher chlorophyll content resulted in higher photosynthetic capacity and overall, increased growth [16]. Increased maize biomass upon *Trichoderma* sp. treatment was also described [5].

In addition to these effects, *Trichoderma* species can also colonize the plants. Biocontrol *Trichoderma harzianum* SQR-T037 and E5 strains were re-isolated from the root and stem of cucumber up to 30 days after root treatment [17]. In tomato, the colonization of internal tissues was detected eight weeks after dipping the roots into *T. harzianum* spore suspension [18]. In the case of perennial plants, re-isolation of a *Trichoderma atroviride* strain (T-15603.1) was demonstrated from treated wounds up to 30 months after the application of the angiosperm (flowering) urban trees [19]. However, the colonization potential of *Trichoderma* can vary on the species and even strain level, as demonstrated on eucalyptus seedlings [20]. In cocoa, *Trichoderma ovalisporum* isolates were able to colonize only the unhardened shoot region, but not the hardened woody stem tissues of the seedlings [21], while *Trichoderma asperellum* strains were re-isolated from root and stem sections one month after inoculation through the roots [22].

The colonization potential of commercial and local *Trichoderma* strains was also studied in grapevine nurseries. *Trichoderma* spp. could effectively colonize wounds [23], and they were re-isolated from the proximity of the pruning wound 21 days after treatment of detached canes [24]. In the case of injury inoculation of grafted plants, *Trichoderma* sp. was detected only near the injury [25]. In field studies, grapevine pruning wound colonization was described with commercial *T. asperellum* ICC 012 and *T. gamsii* ICC 080 strains in grapevine canes up to seven months after the spraying of fresh prunes [23]. In the case of trunk wound inoculation of old grapevine, re-isolation was successful up to 20 months after inoculation with *T. atroviride* (previously *T. harzianum*) strain AG1, and whole-trunk colonization was also described [26]. *T. harzianum, T. atroviride* and *Trichoderma virens* strains were detected in grapevine planting material following graft treatments and drenching, with an up-to-seven-month-long colonization of the basal ends and roots [27].

*Trichoderma*-based products have been demonstrated to be effective biocontrol agents (BCAs) against grapevine trunk diseases (GTDs) both in planta and in field experiments [23,28,29,30]. *T. afroharzianum* T22 (formerly: *T. harzianum* Rifai) and *T. atroviride* SC1 strains—currently the most important biocontrol fungi in grapevine nurseries and the vineyards—promoted the rooting of grafts, and suppressed GTDs [29,31]. Their efficacy has also been demonstrated against other foliar and bunch diseases [32,33,34]. In contrast, much less is known about the biostimulation effect of *Trichoderma* treatment on grapevine growth and yield. Such an effect on root development has been reported by Di Marco and Osti [35]. Additionally, Pascale et al. [36] detected increased yield following *T. harzianum* treatments, and some quality indicators such as polyphenol content and antioxidant activity also increased.

We recently described and characterized endophytic *Trichoderma* strains from grapevines [37]. In this paper we report and discuss the ability of two different mixes of some of these isolates (*T. simmonsii, T. orientale* and *T. gamsii*, as well as *T. afroharzianum* and *T. simmonsii*) for the long-term colonization of plants and stimulatory potential on different grapevine cultivars in commercial vineyards. The stimulatory potential was analyzed in terms of percentage of lost plants (i.e., the percentage of grafts that died after plantation), bud vigor, grape yield, as well as must quality. 

## 2. Materials and Methods

### 2.1. Trichoderma Isolates and Inocula

**Figure 1:**
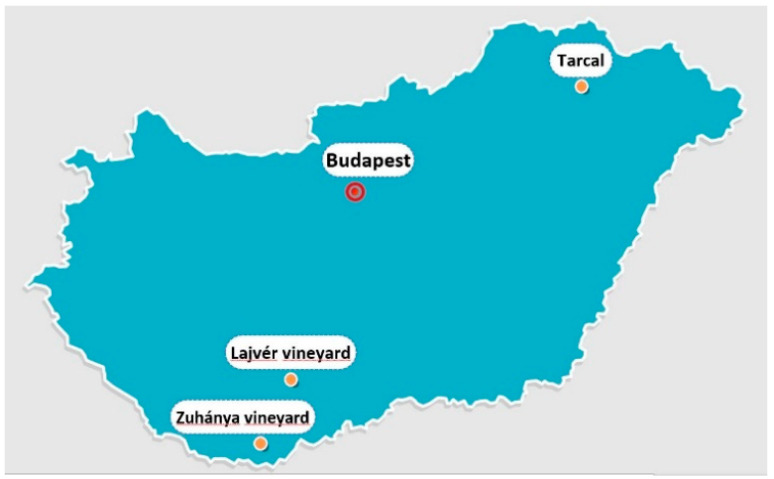
The origin of *Trichoderma* strains (Tarcal), and the location of the experimental fields in Hungary. Zuhánya vineyard correspond respectively to the Experimental field I in the Villány Vine Region, and the Lajvér vineyard Experimental field II in the Szekszárd Vine Region.

Endophytic *Trichoderma* strains were isolated from white grapevine plants (Furmint cultivar) from the Tokaj Wine Region, Northeast Hungary (Figure 1). The isolation and characterization of the *T. afroharzianum* (TR04), *T. simmonsii* (TR05)*, T. orientale* (TR06), and *T. gamsii* (TR08) strains that were used in this study were previously described by Kovács et al. [37]. 

Two different methods were used for inoculum production. In Experimental field I, the inoculum was prepared on Petri dishes (diameter 60 mm) containing potato dextrose agar (PDA, Scharlau, Barcelona, Spain) that were inoculated by dispersing 100 L of conidial suspension (10^5^ spores mL^−1^) on the surface with a sterilized spreader and incubated at room temperature for 5 days. Spores were collected from 7-day-old cultures by twice pipetting 2 mL of sterile distilled water containing 0.01% Tween^®^ 20 (Merck, Budapest, Hungary). Conidial suspensions concentrated at 10^9^ spores mL^−1^ were finally obtained and stored at 4 °C for 3 weeks (Table 1). Spore concentration was determined with a Thoma chamber. Viability was checked by colony-forming unit (CFU) determination after spreading 100 L from different dilutions of the stored solution on PDA plates, as previously described.

The *Trichoderma* conidiospores to be used in Experimental field II were mass-produced by means of submerged bioreactor cultivations. They were carried out in a 9 L scale glass bioreactor (Inel Ltd., Budapest, Hungary) with a culture (working) volume of 6 L, equipped with two six-blade Rushton disc turbine impellers. Operating conditions were pH 5.6, 30 Merck, and 0.5 vvm (volumes of air per volume of liquid per minute). To minimize medium loss, the waste gas from the headspace was cooled in a reflux condenser connected to an external cooling bath (4 ℃) before exiting the system. The growth medium contained 5 g L^−1^ D-glucose, 2.8 g L^−1^ yeast extract, 1 g L^−1^ KH_2_PO_4_, 0.5 g L^−1^ MgSO_4_ × 7H_2_O, 0.01 g L^−1^ FeSO_4_ × 7H_2_O, 0.01 g L^−1^ ZnSO_4_ × 7H_2_O, 0.005 g L^−1^ CuSO_4_ × 5H_2_O and 0.5 g L^−1^ KCl. Sulphate salts and D-glucose were sterilized separately. The bioreactor was inoculated with 100 mL of spore solution of 0.6–1.25 × 10^8^ conidiospores mL^−1^ for each strain, separately (Table 1). Spore concentration as well as culture sterility were checked with an optical microscope. Fermentations were halted on the 4th day, and growth media containing 6.7–4.3 × 10^7^ spores mL^−1^ were stored at 5 ℃. Viability of the conidiospores was checked monthly by CFU determination, performed on PDA plates, as described previously. No decrease in CFUs was detected during storage.

### 2.2. Experimental Fields and Treatments

The experiment was set in two commercial vineyards in the southwest part of Hungary in the Villány Wine region (Experimental field I) and in the Szekszárd Wine region (Experimental Field II) (Figure 1). Their climate characteristics are similar [38]. Experimental Field I is in the Villány Wine Region, in the Zuhánya vineyard (Solum Borbirtok Ltd.) GPS: N 45° 52.398’, E 018° 18.871’, with nearly 12,000 plants. The grafts were soaked in 300 batches for 12–36 h at a temperature of approximately 10 °C. One-year-old rooted grafts with different scion/graft combinations were used (Table 2). 

*Trichoderma* spore suspension was diluted in water by mixing the three different strains (*T. simmonsii*, *T. orientale* and *T. gamsii*) and setting the final concentration to 10^6^ spores mL^−1^ (Table 1). The plants were soaked overnight in order that only the rootstock contacted the *Trichoderma*-containing solution. Control plants were similarly soaked in water without spores. Different cultivars were soaked separately. Plants were planted using hydro-drill following soaking on the 30th of April and the 1st of May 2015. Treated plants were planted in different rows. Sampled treated and non-treated plants were at a minimum of one row distance.

**Figure 2:**
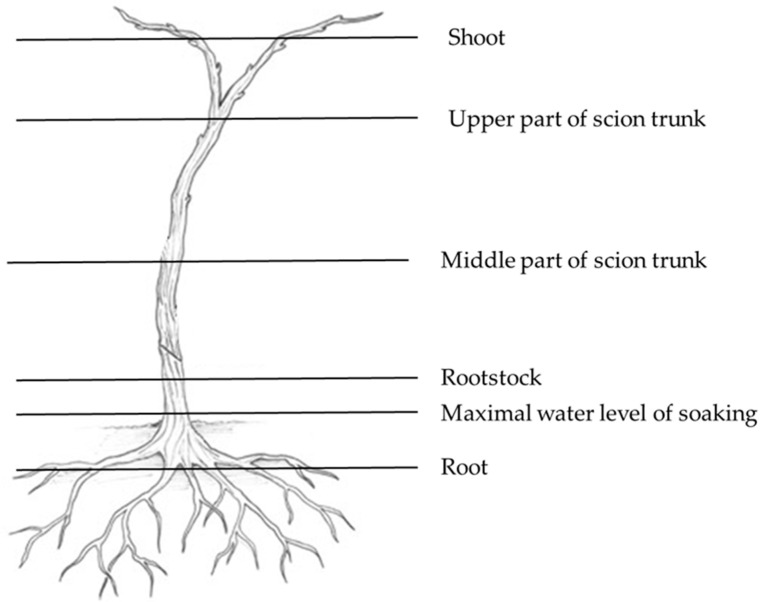
Sampling points for *Trichoderma* spp. re-isolation on grafted vine. Modified drawing of Jason Stafne from [40].

Experimental field II is in the Szekszárd Wine Region, in the Lajvér vineyard (Lajvér Borház Ltd. Szálka, Hungary) (Figure 1) GPS: N 46° 17.694’, E 018° 38.879’. The experiment was set between the 2nd and 4th May 2017, using *T. afroharzianum* and *T. simmonsii* strains (Table 2). Spore solutions were diluted with well water to 10^6^ mL^−1^. The plants were soaked in 300 batches for 48–72 h at a temperature of approximately 10 °C. The plants were soaked and planted as previously described (Figure 2). Treated plants were planted in different terraces. 

Experimental fields were not treated with *Trichoderma* strains in the following period. 

The training system was in a unilateral cordon of medium height in both vineyards.

The same technologies were applied in the experimental fields for the *Trichoderma*-treated and control plants, including 10–12 spraying per year with pesticides. Foliar fertilizer Harvest K (Milagro, Budapest, Hungary) containing potassium was used six weeks before harvest. 

### 2.3. Isolation and Identification of Fungi from Woody Parts of Grapevine Plants

The method previously described in Kovács at al. [37] was followed for the isolation of fungi from the inner, woody part of the plants, as well as from different parts of the grapevine plants (Figure 2). 

After soaking, some of the plants were transferred to the laboratory at the University of Debrecen under chilled conditions to test for the presence of *Trichoderma* spp. In the time that elapsed since planting, samples were taken annually to confirm the presence/absence of *Trichoderma* strains in the treated plants and the untreated ones from both Experimental field I and II. Two controls and four treated samples were taken at each sampling. Moreover, four plants were removed five months after the *Trichoderma* treatment from the Experimental field II and were sent to the University of Debrecen. Plants were cut to allow samplings from different parts (Figure 2). Fungi were isolated from woody trunk as described previously [37]. *Trichoderma* isolates were purified to get monospore isolates for DNA extraction and PCR amplification of *tef1* and ITS region as described previously [37]. 

Identification was based on morphological characteristics as well as on ITS or *tef1* sequences. DNA isolation, amplification and sequencing were performed as described by Kovács et al. [37]. Sequences were deposited in GenBank (ON931231–ON931232 and ON937623–ON937629).

### 2.4. Determination of Lost Plant Percentage

The percentage of lost plants was determined in three years (2018, 2019 and 2021) in Experimental field I. In 2018, the full vineyard was surveyed and the percentage of loss in the rows was used for statistics. In 2019 and 2021, some randomly determined rows were surveyed on the individual level and 40 plants per block were used as objects in statistical analysis. Since the Experimental field II vineyard is younger, the percentage of lost plants was determined in 2021 only.

### 2.5. Determination of the Effect of Trichoderma Treatment on Bud Burst

A survey was conducted to investigate the effect of the *Trichoderma* treatment on bud burst. The survey was conducted in April 2019, i.e., 4 and 2 years after the *Trichoderma* treatments in the Experimental field I and Experimental field II. Altogether, 98–150 plants were examined from each cultivar, and the number of buds, as well as their development was detected.

Bud burst ratio (BB%) was calculated as indicated in Equation (1):

(1) $$\mathrm{BB}\;\%=\frac{\mathrm{no}.\;\mathrm{buds}\;\mathrm{with}\;\mathrm{detected}\;\mathrm{burst}\;}{\mathrm{total}\;\mathrm{no}.\;\mathrm{of}\;\mathrm{buds}\;\mathrm{in}\;\mathrm{the}\;\mathrm{cane}}\times 100$$

The bud burst vigor was evaluated based on a four-grade scale indicated in Table 3. 

Bud burst vigor index (BBVI%) was calculated as follows (Equation (2)):

(2) $$\mathrm{BBVI}\;\%=\frac{\sum \mathrm{Class}\;\mathrm{frequency}\;x\;\mathrm{score}\;\mathrm{of}\;\mathrm{rating}\;\mathrm{class}\;}{\mathrm{Total}\;\mathrm{number}\;\mathrm{of}\;\mathrm{observations}\times 3\;\left(\mathrm{maximal}\;\mathrm{rating}\;\mathrm{class}\right)}\times 100$$

### 2.6. Determination of Grape Yield

The grape production was measured only in Experimental field I on Blaufraenkisch clones four years after the *Trichoderma* treatment. The weight of the harvested grapes was measured per row with a scale (Demandy TCS-B H45x60, Hungary Mérleg Ltd., Budapest, Hungary). 

The experimental yield per plant was calculated according to Equation (3):

(3) $$\mathrm{Experimental}\;\mathrm{yield}\;=\frac{\mathrm{weight}\;\mathrm{of}\;\mathrm{harvested}\;\mathrm{grapes}}{\mathrm{no}.\;\mathrm{planted}\;\mathrm{grapevines}}$$

The potential yield per plant was calculated according to Equation (4):

(4) $$\mathrm{Potential}\;\mathrm{yield}\;=\frac{\mathrm{weight}\;\mathrm{of}\;\mathrm{harvested}\;\mathrm{grapes}\;}{\mathrm{no}.\;\mathrm{living}\;\mathrm{grapevines}}$$

The potential yield represents an ideal yield in a plantation without the loss of plants, where GTDs are not present

### 2.7. Determination of Chemical Parameters from the Must

Must (freshly crushed berry juice, containing seeds, skins and stems) was sampled in Experimental field II in 2019. The examined musts represented a mixed, aggregate sample of two control and two rows treated with a combination of *T. afroharzianum* and *T. simmonsii*. Must parameters were determined with FOSS WineScan Flex (Agromilk Analitica Ltd., Szeged, Hungary) following the producer’s description. Data were calculated from two measurements with less than a 5% difference in between.

### 2.8. Statistical Analysis

The mean ratios of lost plants, bud burst ratio (BB%), bud burst vigor index (BBVI%) and the yield of the control and the *Trichoderma* treated groups were compared with non-parametric Mann–Whitney U-test, since our data did not fulfill the assumptions of parametric tests: normality and homogeneity of variances. The normality was tested with Q-Q plots, while in the case of homogeneity of variances, the Levene test was used. The differences in the qualitative parameters of the must of the control and treated groups were provided with percentages because only two samples were measured per group. Analysis was carried out with Statistica 7 program package.

## 3. Results

### 3.1. Potential Grape Wood Colonization by Trichoderma spp.

Different grapevine cultivars in two commercial vineyards were used to test the effects of previously characterized endophytic *Trichoderma* strains [37]. The grafted plants were treated before planting (see Materials and Methods for details) and sampled afterwards to detect the colonization potential of the strains.

No *Trichoderma* was detected in the woody parts of any of the control grapevine. However, *T. afroharzianum*, *T. orientale and T. gamsii* strains could be cultured and identified—based on *tef1* or ITS sequence similarities—from some of the treated plants, not only from the root but also from the trunk of the scion (Table 4). *T. afroharzianum* was re-isolated from different parts of the same plant (root or rootstock and scion).

The presence of *T. afroharzianum*, *T. orientale and T. gamsii* could be confirmed several months and even years after the treatments (Table 4). Two of the three strains (*T. orientale and T. gamsii*) could be identified from Experimental field I after their application, while one of the two applied *Trichoderma* strains (*T. afroharzianum*) could be identified from Experimental field II five months after the treatment (Table 4). 

### 3.2. Loss of Planted Grafts

Comparing the *Trichoderma*-treated plants with the controls, no statistically significant differences were detected for the loss of planted grafts in the 3–4 years that followed planting in either experimental field, based on the Mann–Whitney U-test (Table 5). However, the overall loss tended to be reduced in *Trichoderma*-treated plants. In particular, the highest loss of plants (9.46%) was detected in the non-treated Cabernet Sauvignon cultivar in Experimental field II, four years after planting. The *Trichoderma* treatment more than halved this loss (4.38%). A similar result was detected on a Cabernet Franc cultivar in Experimental field I, where the treatment decreased the loss of planted grafts from 1.88% to 0.78%. 

### 3.3. Bud Burst Ratio and Vigor Index

**Figure 3:**
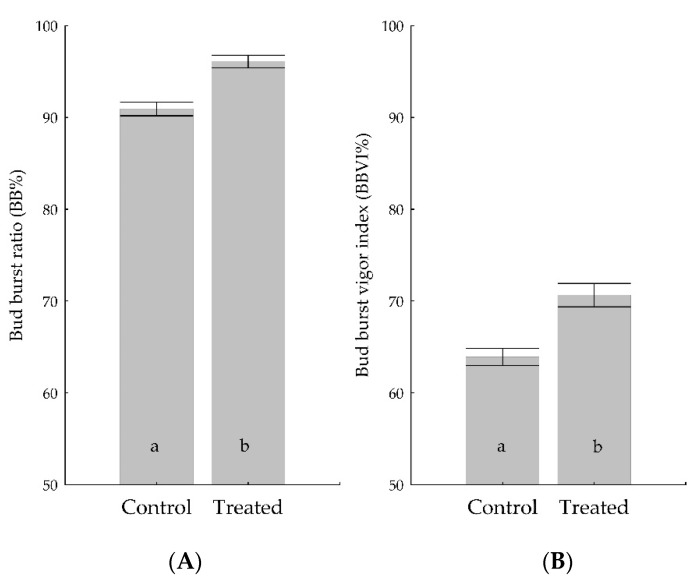
Effect of *Trichoderma* treatment (Treated) on bud burst (**A**) and bud burst vigor index (**B**) values for the whole sample. *Trichoderma* treatment with combination of *T*. *simmonsii*, *T. orientale* and *T*. *gamsii*, or *T*. *afroharzianum* and *T. simmonsii*. Small letters show significant differences between treatments based on Mann–Whitney U-test (*p* < 0.05).

The positive effect of *Trichoderma* was detected up to four years after the root-soaking treatment with the spore suspension. Analyzing data from both experimental fields, including all cultivars and clones, the *Trichoderma* treatment significantly increased both the bud burst ratio (BB %) and the bud burst vigor index (BBVI %) (Figure 3).

At the time of the survey, the majority of the buds were out of dormancy and were between the start of bud swelling and leaf development. The average bud burst ratio was higher (*p* < 0.001) in the plants treated with *Trichoderma* compared to the controls (Figure 3A). The average bud burst vigor index was also higher (*p* < 0.001) in the plants previously treated with *Trichoderma* than in the controls (Figure 3B). The BB% of the treated plants increased in most of the cultivars by over 5% (Table 6). The BBVI% also increased in all but one case (Table 6). The *Trichoderma* treatment in the Kt1 clone of Blaufraenkisch cultivar resulted in a higher BB%, but a lower BBVI% (Table 6). This result may indicate that although more buds started to develop, their vigor was lower, possibly due to the restricted nutrient supply capacity of the 5BB (K21) rootstock.

The Cabernet Sauvignon cultivars exhibited extremely high BB% and BBVI% values independently of the *Trichoderma* treatment; thus, while the treated grapevine still showed higher values, the differences were not statistically significant (Table 6). These high BB% values may have been due to a vineyard management technology fitting for this particular cultivar. 

### 3.4. Quantity and Quality of Harvested Grapes

The grape production was measured only in Experimental field I on Blaufraenkisch clones four years after the *Trichoderma* treatment. Comparing the *Trichoderma*-treated plants with the control, no statistically significant differences were detected for the experimental, nor for the potential yield per plant, based on the Mann–Whitney U-test (*p* = 0.3488 and 0.3672, respectively, Table S1). However, it is possible to note that overall, the *Trichoderma* treatment slightly increased these parameters by 12.2% and 13.27%, respectively, compared to the control (Table S1).

To assess the possible effect of the *Trichoderma* treatment on the must quality, some oenological parameters were determined in the freshly crushed grape juice (must). There were only minimal differences in pH, total acidity and glucose/fructose ratio following *Trichoderma* treatment (Table 7). Glycerol and volatile acidity concentrations were low (<0.9 and <0.12 g L^−1^, respectively). However, the values of Brix, as well as the extract concentrations—the reducing sugar, D-glucose and D-fructose—tended to be increased by the *Trichoderma* treatment compared to the control, reaching 3.7%, 4.64%, 4.35%, 4.79% and 3.7%, respectively, indicating a potentially higher ethanol content for the produced wine. Potassium concentration was also 3.45 % higher.

## 4. Discussion

In this study we demonstrated that grapevine-endophytic *Trichoderma* strains could colonize Blaufraenkisch, Cabernet Franc and Cabernet Sauvignon cultivars for up to four years after the treatment. Whole plant colonization capability of the strains was detected within five month following the treatment. By “treatment”, drenching of the grafted grapevines into *Trichoderma* spp. spore suspension before planting is meant. *Trichoderma* strains were originally isolated from white grape cultivars from the Tokaj Wine Region in the northeastern part of Hungary, some 400 km away from the two commercial vineyards in southwest Hungary where this study took place [37]. This physical distance between the sites of the isolation and biostimulation experiments effectively excluded any cross-contaminations. The longest reported period of grapevine colonization by *Trichoderma* species in nurseries was only seven months [27]. Our results imply long-term, whole-plant colonization potential for these endophytic *Trichoderma* strains in spite of competitive microbiota and regular fungicide applications. 

Due to their mycoparasitic, antifungal and plant-defense-inducing properties, *Trichoderma* applications typically target fungal pathogens, and thus they are defined as biocontrol agents [23,28,31]. In contrast, information is limited on their stimulatory effect in general and their enhancement of grapevine growth and production in particular, although *Trichoderma* applications were described to increase the root area and the percentage of certifiable vines in nurseries [35]. Different stress factors may result in the loss of plants in young plantations, such as nursery induced stress, vineyard establishment and management stresses (e.g., nutritional deficiency or toxicity and frost damage), and biological stresses, such as GTD pathogens [42]. Adequate root systems are important to provide nutrients and cope with environmental or biotic stress. Therefore, a well-developed root system contributes to the mitigation of young vine decline in vineyards [42]. In the present study, the loss of plants in vineyards following pre-planting treatment with either a mix of the endophytic *Trichoderma* strains (i.e., *T. simmonsii*, *T. orientale* and *T. gamsii*, or *T. afroharzianum* and *T. simmonsii*) was not statistically significantly affected by the *Trichoderma* treatment, even if a tendency to be reduced has been highlighted. The effect seems to be cultivar-dependent, but we cannot explain the cause at the moment. 

We also detected a biostimulatory effect in the form of enhanced bud development, as both the average bud burst ratio (i.e., percentage of buds being out of dormancy) and the bud burst vigor index were significantly higher in plants treated with *Trichoderma* spp., relative to the control. Again, there were differences between the cultivars as well as the clones. Among the mechanisms underlying plant growth stimulation, the importance of fungal siderophores resulting in increased iron uptake has been demonstrated [10,43,44]. Production of the plant hormone auxin by *Trichoderma* fungi as well as a special fungal cell wall protein may also stimulate growth of the root and other parts of the plant [45,46]. Increased photosynthetic activity after endophytic *Trichoderma* spp. treatments [47] may as well increase the plant growth, production [36,48] and sugar content of the fruits [48].

The yields and quality of grape production is strongly influenced by the number and the distribution of viable shoots on a cane [49]. However, extreme weather conditions in the early phenological stages, as well as various forms of biotic damage may decrease the viability of the overwintered buds. Increased stress tolerance against different abiotic factors such as drought was reported following *Trichoderma* treatments [50]. The increasing stress tolerance following *Trichoderma* treatments was explained by more efficient plant responses to free oxygen radicals [51,52]. Besides the climatic factors, various forms of biotic damage may also decrease the viability of the overwintered buds, such as mites, insects and fungal trunk diseases [53,54,55]. The latter have been playing an increasingly important role worldwide since the availability of chemical management techniques is restricted [29,31]. As demonstrated in this study, the *Trichoderma* treatment of the grafts could have a positive effect on the overwintering and bursting of buds together with the shoot development. The biostimulatory and conditioning role in the previous year can support the differentiation and cold resistance of the buds. Additionally, the plant colonization by *Trichoderma* can exert biocontrol activity, by controlling or suppressing the pathogens in the vessels and woody tissues [25], thereby the nutrient supply of the buds may remain uninterrupted, which is crucial for grapevine vitality [52]. Moreover, the biostimulatory effect may also result in a better bursting vigor and acceleration of bud and shoot development. Even if it is often difficult to distinguish the biocontrol (antifungal) and biostimulatory effects of *Trichoderma* isolates and inocula, our results suggest that the treatment of the grafts with a mix of selected *Trichoderma* strains can positively contribute to grapevine vitality. Besides enhanced bud development, <10% more grapes from the *Trichoderma*-treated plants could be harvested, with the sugar content also tending to increase. 

In conclusion, this study conducted in commercial vineyards shows that the graft treatment with mixes of selected endophytic *Trichoderma* strains can both long-term colonize the grapevine plants and exert a stimulatory effect on the buds. Further studies are needed to confirm the tendency to reduce the young plant decline after planting and increase the grape yield as well as improve the must quality. 

## 5. Patents

Authors ES and KCs are the inventors of a Hungarian patent entitled, Biopeszticid gombatörzsek és készítmények”. P1800012, Submission Year: 2018, NSZO: C12R 1/885, A01N 63/00, A01N 63/04, C12N 1/00. The patent provides legal protection to the biocontrol-related applications of some of the *Trichoderma* strains described in this manuscript in Hungary.

## Figures and Tables

**Table 1:** *Trichoderma* inocula used in the different experimental fields.

Experimental Field	*Trichoderma* Strains	Inoculum Production Method	Spore Concentration (Spores mL^−1^)	
			following Production	in Soaking Treatments
I (Siklós, Zuhánya)	*T. simmonsii* (TR05)	Culture on PDA medium	1.5 × 10^9^	10^6^
	*T. orientale* (TR06)		1.1 × 10^9^	
	*T. gamsii* (TR08)		1.4 × 10^9^	
II (Szálka, Lajvér)	*T. afroharzianum* (TR04)	Submerged liquid culture	4.3 × 10^7^	10^6^
	*T. simmonsii* (TR05)		6.7 × 10^7^	

**Table 2:** Grapevine scion-rootstock combinations used for *Trichoderma* treatment.

Experimental Field	Cultivar (Clone) *	Rootstock (Clone)	Planting Time
I (Siklós, Zuhánya)	Blaufraenkisch (Kt.1.)	5BB (K21)	17.04.–20.04.2015
	Blaufraenkisch (A4/1)	5BB (We48)	
	Cabernet Sauvignon (337)	K5BB (ISV1)	
	Cabernet Franc (GM/Trv)	K5BB (101)	
	Cabernet Franc (E11)	K5BB (ISV1)	
	Cabernet Franc (ISV5)	K5BB (GM13)	
II (Szálka, Lajvér)	Cabernet Sauvignon (E153)	K5BB (ISV1)	02.05.–04.05.2017

* The nomenclature of grape cultivars is based on the VIVC (Vitis International Variety Catalogue) database [39].

**Table 3:** Bud burst vigor scale applied in bud development survey.

Scale	BBCH Value *	Description of Phenological Stage
0	0	The winter buds are dormant or aborted
1	01–05	Start of bud swelling to “wool stage”
2	07–09	Bud burst
3	11–15	Starting of leaf development

* BBCH: scale to identify the phenological development stages of plants [41].

**Table 4:** Re-isolated *Trichoderma* strains from Experimental fields I and II.

Experimental Field	ID of Isolates	Identified *Trichoderma* Strain	ID of Sequences	Plant Part of Re-Isolation	Date of Sampling for Re-Isolation	Elapsed Time between Treatment and Re-Isolation
I.	RIV3	*T. orientale*	ON937623	scion trunk (upper part)	October 2016	15 months
	RIV6		ON937624	scion trunk (upper part)	October 2016	15 months
	RIV7		ON937625	scion trunk (upper part)	October 2016	15 months
	RIV41	*T. gamsii*	ON931231	root	August 2019	4 years
	RIV42		ON931232	root	August 2019	4 years
II.	RIS1	*T. afroharzianum*	ON937626	root	October 2017	5 months
	RIS2		ON937627	rootstock	October 2017	5 months
	RIS5		ON937628	root	October 2017	5 months
	RIS6		ON937629	scion trunk (middle part)	October 2017	5 months

**Table 5:** Effect of *Trichoderma* treatment (*Trichoderma*) on lost plants (%; mean ± SE) of the whole sample and by cultivars.

Cultivar	Experimental Field	n ^1^	U-Test ^2^	Loss of Planted Grafts (%)	
				Control	*Trichoderma* ^3^
All	I and II	68	*p* = 0.4945	5.33 ± 1.12	3.71 ± 0.54
Cabernet Franc	I	16	*p* = 0.1770	1.88 ± 0.60	0.78 ± 0.36
Blaufraenkisch	I	30	*p* = 0.1187	3.17 ± 0.68	4.83 ± 0.74
Cabernet Sauvignon	II	22	*p* = 0.0943	9.46 ± 2.46	4.38 ± 1.03

^1^ n: number of statistical samples. ^2^: Mann–Whitney U-test. ^3^: *Trichoderma* treatment (*Trichoderma*) with combination of *T. simmonsii*, *T. orientale* and *T. gamsii*, (Experimental field I) or *T. afroharzianum* and *T. simmonsii* (Experimental field II).

**Table 6:** Effect of *Trichoderma* treatment on mean bud burst ratio (BB%) and bud burst vigor index (BBVI%) values of different cultivars and clones, in the two experimental fields.

Cultivar	Experimental Field	Clone	n ^1^	Treatment ^2^	BB% ± SE ^3^	BBVI% ± SE ^3^
Cabernet Franc	I	E11	149	Control	94.97 ± 1.10a	69.36 ± 1.83a
				*Trichoderma*	99.60 ± 0.40b	90.74 ± 1.78b
	I	ISV5	98	Control	86.29 ± 2.52+	52.13 ± 2.26+
				*Trichoderma*	92.31 ± 1.98+	57.54 ± 2.22+
	I	N101	142	Control	91.74 ± 1.48+	64.41 ± 1.95a
				*Trichoderma*	96.50 ± 1.16+	71.05 ± 2.09b
	I	all	389	Control	91.96 ± 0.91a	63.96 ± 1.23a
				*Trichoderma*	96.15 ± 0.81b	73.28 ± 1.64b
Blaufraenkisch	I	A4	150	Control	86.48 ± 1.98a	52.49 ± 1.76a
				*Trichoderma*	92.43 ± 2.41b	60.83 ± 2.32b
	I	Kt1	147	Control	88.96 ± 1.85a	67.41 ± 2.08b
				*Trichoderma*	95.60 ± 2.31b	60.83 ± 4.11a
	I	all		Control	87.72 ± 1.35a	59.95 ± 1.46
				*Trichoderma*	93.97 ± 1.67b	60.83 ± 2.31
Cabernet Sauvignon	II	E153	98	Control	98.96 ± 1.04	80.21 ± 2.49
				*Trichoderma*	100.00 ± 0.00	82.11 ± 2.49

^1^ n: number of surveyed plants. ^2^: *Trichoderma* treatment: combination of *T. simmonsii*, *T. orientale* and *T. gamsii* (Experimental field I), or *T. afroharzianum* and *T. simmonsii* (Experimental field II)). ^3^: means with different letters differ significantly based on Mann–Whitney U-test (*p* < 0.05), and “+” indicates differences at *p* < 0.1 level. SE: standard error.

**Table 7:** Quality parameters determined from must of harvested Cabernet Sauvignon grapes with and without *Trichoderma* treatment.

Must Parameter	Control	*Trichoderma* ^1^
Brix (°Bx)	22.80	23.70
Extract (g L^−1^)	256.95	268.10
Reducing sugar (g L^−1^)	230.50	241.20
Glucose (g L^−1^)	109.65	114.90
Fructose (g L^−1^)	117.50	121.85
Glycerol (g L^−1^)	0.60	0.80
pH	3.39	3.42
Total Acidity (g L^−1^)	6.30	6.40
Volatile Acidity (g L^−1^)	0.09	0.11
Potassium (mg L^−1^)	1114.50	1153.00

^1^ Two years after *Trichoderma* treatment with combination of *T. afroharzianum* and *T. simmonsii*.

## Data Availability

The datasets used in the current study are available from the corresponding author on reasonable request.

## Supplementary Materials

The following are available online at https://www.mdpi.com/article/10.3390/pathogens12010002/s1, Table S1: Effect of the Trichoderma treatment (*Trichoderma*) on the yield of Blaufraenkisch clones in the Experimental field I.
