# Supplementary material for: The Biocontrol Potential of Endophytic Trichoderma Fungi Isolated from Hungarian Grapevines, Part II, Grapevine Stimulation
Source: Pathogens. 2022 Dec 20;12(1):2. doi: 10.3390/pathogens12010002 (PMC9863551; doi:10.3390/pathogens12010002)
Supplement: Supplementary file 1 [file pathogens-12-00002-s001.zip › pathogens-1952272-supplementary.pdf]

Table S1. Effect of the *Trichoderma* treatment (*Trichoderma*) on the yield of Blaufraenkisch clones in the Experimental field I.

Blaufraenkisch clone	Treatment[1]	Row	No. of grapevines	Plant loss (%)	Yield (kg/plant)	
					experimental	potential
A4/1	Control	1	148	2.70	2.68	2.76
		2	165	4.24	2.78	2.90
		3	165	1.82	2.22	2.26
		4	165	1.21	3.15	3.18
		5	165	1.21	1.57	1.59
	mean(±SE)			2.24(±0.57)	2.48(±0.27)	2.54(±0.28)
	Trichoderma	1	165	4.24	3.15	3.28
		2	165	2.42	2.78	2.84
		3	165	2.42	2.96	3.03
		4	165	0.61	2.59	2.60
		5	165	1.21	2.41	2.44
		6	165	1.82	2.59	2.64
	mean(±SE)			2.12(±0.51)	2.74(±0.11)	2.81(±0.13)
Kt1	Control	1	187	9.09	2.45	2.69
		2	187	5.88	2.28	2.43
		3	187	0.53	1.47	1.48
		4	187	5.88	1.30	1.39
	mean(±SE)			5.35(±1.77)	1.88(±0.29)	2.00(±0.33)
	Trichoderma	1	187	8.56	2.28	2.50
		2	187	8.56	2.28	2.50
		3	187	3.74	2.45	2.54
		4	187	8.02	1.80	1.95
		5	187	10.16	1.96	2.18
		6	187	7.49	2.12	2.29
		7	187	3.21	2.94	3.03
	mean(±SE)			7.10(±0.99)	2.26(±0.14)	2.43(±0.13)
Overall mean	Control			3.62(±0.96)	2.21(±0.21)	2.30(±0.22)
	Trichoderma			4.80(±0.91)	2.48(±0.11)	2.60(±0.10)

[1]: treatment (*Trichoderma*) with combination of *T. simmonsii*, *T. orientale* and *T. gamsii*
